# The association of healthy lifestyle score and risk of non-alcoholic fatty liver disease

**DOI:** 10.1186/s12889-023-15816-3

**Published:** 2023-05-26

**Authors:** Mitra Kazemi Jahromi, Ghazal Daftari, Hossein Farhadnejad, Asal Neshatbini Tehrani, Farshad Teymoori, Ammar Salehi-Sahlabadi, Parvin Mirmiran

**Affiliations:** 1 Endocrinology and Metabolism Research Center, Hormozgan University of Medical Sciences, Bandar Abbas, Iran; 2 School of Medicine, Tehran University of Medical Sciences, Tehran, Iran; 3 Nutrition and Endocrine Research Center, Research Institute for Endocrine Sciences, Shahid Beheshti University of Medical Sciences, Tehran, Iran; 4 Student Research Committee, Ahvaz Jundishapur University of Medical Sciences, Ahvaz, Iran; 5 Department of Nutrition, School of Allied Medical Sciences, Ahvaz Jundishapur University of Medical Sciences, Ahvaz, Iran; 6 Department of Nutrition, School of Public Health, Iran University of Medical Sciences, Tehran, Iran; 7 Department of Clinical Nutrition and Dietetics, Faculty of Nutrition and Food Technology, National Nutrition and Food Technology Research Institute, Shahid Beheshti University of Medical Sciences, Tehran, Iran; 8 Department of Community Nutrition, School of Nutrition and Food Science, Isfahan University of Medical Sciences, Isfahan, Iran

**Keywords:** Dietary pattern; lifestyle, Healthy lifestyle score, Alternate healthy eating index, Non-alcoholic fatty liver disease; adults

## Abstract

**Background:**

The combined role of important environmental factors as a single lifestyle index in predicting non-alcoholic fatty liver disease (NAFLD) risk is not fully assessed. Therefore, we aimed to investigate the association of healthy lifestyle factor score (HLS) with the odds of NAFLD in Iranian adults.

**Methods:**

This case-control study was conducted on 675 participants, aged ≥ 20–60 years, including 225 new NAFLD cases and 450 controls. We measured dietary intake information using a validated food frequency questionnaire and determined diet quality based on the alternate healthy eating index-2010(AHEI-2010). The score of HLS was calculated based on four lifestyle factors, including a healthy diet, normal body weight, non-smoking, and high physical activity. An ultrasound scan of the liver was used to detect NAFLD in participants of the case group. Logistic regression models were used to determine the odds ratios(ORs) and 95% confidence interval(CI) of NAFLD across tertiles of HLS and AHEI.

**Results:**

Mean ± SD age of the participants were 38.13 ± 8.85 years. The Mean ± SD HLS in the case and control groups was 1.55 ± 0.67 and 2.53 ± 0.87, respectively. Also, the Mean ± SD AHEI in the case and control groups was 48.8 ± 7.7 and 54.1 ± 8.1, respectively. Based on the age and sex-adjusted model, the odds of NAFLD were decreased across tertiles of AHEI (OR:0.18;95%CI:0.16–0.29,P_trend_<0.001) and HLS(OR:0.03;95%CI:0.01–0.05,P_trend_<0.001). Also, in the multivariable model, the odds of NAFLD were decreased across tertiles AHEI (OR:0.12;95%CI:0.06–0.24,P_trend_<0.001) and HLS(OR:0.02;95%CI:0.01–0.04,P_trend_<0.001).

**Conclusions:**

Our findings reported that higher adherence to lifestyle with a higher score of HLS was associated with decreased odds of NAFLD. Also, a diet with a high AHEI score can reduce the risk of NAFLD in the adult population.

## Background

During the past four decades, non-alcoholic fatty liver disease (NAFLD) has been the main cause of the chronic fatty liver disease [1], with a strong association with parameters related to metabolic syndrome [2]. Approximately 25% of the global adult population suffers from NAFLD [1]. Mortality related to liver disorders is growing fast worldwide due to the high prevalence of NAFLD [3], and it is considered a critical reason for end-stage liver disease [4]. Multiple factors affect the NAFLD prevalence, such as diagnostic methods and the population in which the study was carried out. Middle East (20–30%) and South America (30%) are on top of the list of NAFLD prevalence [1]. According to a systematic review, the prevalence of NAFLD is estimated at 34% in Iran [5].

Along with genetic predisposition, various environmental factors, including elevated body weight [6], physical inactivity [7], and smoking [8], are suggested as major risk factors for liver damage which play a key role in the occurrence and development of NAFLD. Also, based on the finding of epidemiological studies, higher adherence to a healthy diet rich in whole grains, fruits, vegetables, nuts, and legumes can play a preventive role in reducing the risk of NAFLD; however, an unhealthy diet such as the western style diet, which is characterized by high intake of saturated fat, sodium, and sugar, can accelerate the risk of NAFLD [9]. Different indices have been developed to measure the quality of the diet, one of the most famous of which is the Healthy Eating Index (HEI), which was created based on dietary recommendations for the American population. In 2000, McCullough et al. introduced an alternative version of this index called the Alternative Healthy Eating Index (AHEI) [10]. Previous studies have shown that the ability of AHEI to predict major chronic diseases such as cardiometabolic diseases is significantly higher than HEI [11]. The AHEI score ranges from 0 (no adherence) to 110 (high adherence) have been shown a diet with a higher AHEI score can predict a reduction in the risk of chronic diseases, such as liver diseases [12]. Therefore, investigating the relationship between this modified and new version of HEI with the NAFLD risk in the Middle East and North Africa region, such as Iranian population, can help to confirm the evidence reported on the possible role of AHEI in reducing the risk of NAFLD.

Considering the independent possible role of each of the above-mentioned environmental factors in predicting the risk of liver disease, investigating the combined role of these factors can be more helpful in predicting the risk of NAFLD. Indeed, although in previous studies [13–15], various factors affect whether or not to develop a pathological condition such as NAFLD, examining each of these factors in isolation cannot show the real effect of that factor. As a result, it seems that a lifestyle index by combining a number of these factors, taking into account the interactions between them, shows the simultaneous effect of different factors on the disease in question, which is closer to reality. Recently the combined role of healthy lifestyle score (HLS), defined by a healthy diet (assessed by AHEI), not smoking, no obesity, and vigorous physical activity, as a single variable, in reducing the risk of chronic diseases such as coronary disease [16], type 2 diabetes [17], metabolic syndrome [18], and mortality [19]. Also, some studies recently conducted in Western countries [20, 21] or Chinese [22, 23] reported that higher adherence to lifestyle with a high score of HLS can be associated with reduced risk of NAFLD or liver dysfunction. However, there is no study on the relationship between HLS and the risk of NAFLD in the MENA region, such as the Iranian population.

Considering that the prevalence of NAFLD is relatively high among Iranian adults [5]. Also, to the best of our knowledge, there is no study investigating the possible link between the HLS score and the risk of NAFLD in Iranian adults, which may have a different lifestyle and eating habits compared to Western countries, therefore, we aimed to assess the association of the higher score of healthy lifestyle factors on prediction of NAFLD risk.

## Methods

### Study population

The present study was conducted at the Metabolic Liver Disease Research Center, a referral center affiliated with Isfahan University of Medical Sciences. Among the patients referred to have an Ultrasonography test on their liver and bile ducts because of an abnormal or slight elevation in liver enzymes, dyslipidemia, or being at risk of metabolic syndrome or metabolic syndrome, etc. NAFLD status (yes or no) of patients was ascertained using the results of the Ultrasonography test and endocrinologist visit. Then using a convenience sampling method for selecting cases and controls, we invited NAFLD patients as cases and non-NAFLD as controls to participate in our study if they were willing. The study details were previously reported in our published paper [24]. We entered the 225 newly diagnosed NAFLD patients and 450 controls into our study based on the following inclusion criteria: participants aged 20–60 years who had no special diet (due to a particular disease or weight loss), no history of renal and hepatic diseases (Wilson’s disease, autoimmune liver disease, hemochromatosis, virus infection, and alcoholic fatty liver), CVD, diabetes, malignancy, thyroid disorder, and autoimmune, and not used the potentially hepatotoxic or steatogenic drugs. Our criteria for excluding participants were completing less than 35 items of the food frequency questionnaire and having under or over-reported daily energy intake (≤ 800 or ≥ 4500 kcal/d). All subjects provided written informed consent before the study enrollment.

### Measurements

#### Dietary assessment

Dietary assessment in the present case-control study was conducted by a validated 168-item semi-quantitative food frequency questionnaire (FFQ) [25]. The FFQ consists of a list of typical Iranian foods with standard serving sizes. The consumption frequencies of each food item were put into nine categories. Participants were asked to report their average dietary intake during the previous year by choosing one of the following choices: never or less than once a month, 3–4 times per month, once a week, 2–4 times per week, 5–6 times per week, once daily, 2–3 times per day, 4–5 times per day, and 6 or more times a day. Portion sizes of each food item were converted into grams using standard Iranian household measures [26]. The daily dietary intake of energy and different nutrients for each participant was calculated based on the reported amounts of energy and nutrients in the United States Department of Agriculture’s (USDA) Food Composition Table (FCT) [27]. The Iranian FCT was used for some traditional foods that are not listed in USDA FCT.

#### Anthropometric assessment

A trained dietician measured the participant’s weight using a standard digital Seca scale (made in Germany) while participants wore minimum clothes and without shoes and recorded to the nearest 100 g. Height also was measured using a mounted tape meter in a standing relaxed shoulder position with no shoes to the nearest 0.5 cm. Body mass index (BMI) was calculated as weight (kg) divided by height ($$ {m}^{2}$$).

#### Physical activity assessment

The physical activity data of participants was measured using the International Physical Activity Questionnaire (IPAQ) through face-to-face interviews [28]. All data of the IPAQ were reported as Metabolic Equivalents per week (METs/week) [29, 30].

#### Assessment of other variables

Using a pre-designed demographic questionnaire, information on age, sex, marital status, smoking status, and other variables was obtained. A socioeconomic status (SES) [31] score was calculated based on five dichotomous variables with score points of 0 and 1, including education (academic = 1, non-academic education = 0), family size (≤ 4 people = 1, >4 people = 0), income (high = 1, moderate and low = 0), house acquisition (house ownership = 1, without personal housing = 0), and foreign travel (yes = 1, no = 0). Then, the total SES score was computed by summing up the assigned scores (minimum SES score of 0 to maximum score of 5). Where an SES score of 3, 4, and 5 equated to high, 2 was scored as moderate, and 1 or 0 was scored as low [31].

#### Alternate healthy eating index

The AHEI was developed based on the consumption of 6 food groups as serving sizes (including vegetables, fruits, whole grains, sugar-sweetened beverages and fruit juice, nuts and legumes, and red/processed meat), 4 nutrients (including trans fatty acid, Long-chain (n-3) fats, polyunsaturated fatty acid, and sodium), and alcohol consumption[32]. Regarding the religious prohibition of alcohol consumption in Islamic culture, our population mostly had no consumption of alcoholic beverages, or because of religious taboos, they refused to report it; so we have no data on alcohol consumption and calculated the AHEI using 10 mentioned components. All AHEI-2010 components were scored from 0 (worst) to 10 (best) using the defined criteria in the developed study of AHEI-2010 [32], and the total AHEI-2010 score ranged from 0 (non-adherence) to 100 (perfect adherence) in our study.

#### Healthy lifestyle score

A healthy lifestyle score was computed according to the Patel et al. study [19], which used the four dichotomous components with a point of 0 and 1, including BMI (BMI < 25 kg/m^2^ = 1, BMI ≥ 25 kg/m^2^ = 0), smoking status (non-smoker = 1, smokers = 0), physical activity (higher than median = 1, lower than median = 0), and AHEI score (quintiles of 4 and 5 = 1, quintiles 1, 2, 3 = 0). The points earned for each component were summed and formed the total healthy lifestyle score for each participant. The subject could have a minimum of 0 and a maximum of 4 healthy lifestyle points. Based on the distribution frequency of healthy lifestyle points in our participants, points 0 and 1, points 2, and points 3 and 4, we classified them as tertiles 1, 2, and 3, respectively.

### Statistical analysis

Kolmogorov-Smirnov’s test and histogram char was used to assess the variables’ normality. Participants’ characteristics and dietary intakes were expressed as mean ± standard deviation (SD) or median (25–75 interquartile range) for continuous variables and percentages for categorical variables. The differences in variables between NAFLD and non-NAFLD groups were compared using an independent sample t-test and chi-square for quantitative and qualitative variables, respectively. Study populations were classified into tertiles based on AHEI and healthy lifestyle scores. The demographic and dietary data were presented based on the tertiles of the AHEI index, and P for the trend of continuous and categorical variables across tertiles of the AHEI index was computed using linear regression (median values of AHEI index in each tertile as the independent variable and continuous variables as dependent variable) and chi-square test. The association of the AHEI index and healthy lifestyle score with the odds of NAFLD was assessed using logistic regression analysis. The analysis was adjusted for potential confounders, including age and sex, BMI (only for AHEI), smoking (only for AHEI), physical activity (only for AHEI), marital status, SES, and dietary intake of energy. The odds ratio (OR) with a 95% confidence interval (CI) of NAFLD across tertiles of the AHEI index and healthy lifestyle score was reported. Statistical analysis was conducted using Statistical Package Software for Social Science, version 21 (SPSS Inc., Chicago, IL, USA), and p-values < 0.05 were considered statistically significant.

## Results

The mean ± SD age and BMI of the participants were 38.13 ± 8.85 years and 26.8 ± 4.3 kg/m^2^. The Mean ± SD HLS in the case and control groups was 1.55 ± 0.67 and 2.53 ± 0.87, respectively. Also, the Mean ± SD AHEI in the case and control groups was 48.8 ± 7.7 and 54.1 ± 8.1, respectively.

Table 1 shows study population characteristics among NAFLD patients and healthy participants. Compared to the control group, NAFLD patients are more likely to be smoked, low active, more married, and have higher BMI levels and high SES (P < 0.05). Also, the intakes of energy, red and processed meat, sugar-sweetened beverages and fruit juice, and TFA was higher in NAFLD patients than participants in the control group (P < 0.05), whereas individuals in the case group had lower AHEI and HLS scores and lower vegetable intakes than those in in the control group (P < 0.05). The control and case groups observed no significant difference in other characteristics.

**Table 1:** Study population characteristics among the NAFLD patients and healthy participants^*^

	Non-NAFLD (n = 450)	NAFLD (n = 225)	P-Value
**Demographic data**			
Age,(year)	37.9 ± 8.9	38.6 ± 8.7	0.296
Women, (%)	48.2	44.4	0.354
Body mass index (Kg.m^2^)	25.0 ± 3.1	30.6 ± 4.0	**< 0.001**
Physical activity ( MET/min/week)	1590 ± 949	1119 ± 616	**< 0.001**
Smoking (yes, %)	2.7	7.1	**0.006**
Marital status (married, %)	81.3	88.8	**0.013**
Education level (Bachelor and higher, %)	47.8	44.9	0.478
Socio economic status (%)			**< 0.001**
Low (%)	31.6	21.3	
Middle (%)	40.4	33.8	
High (%)	28.0	33.6	
**Dietary intake**			
Energy intake(Kcal/d)	2227 ± 645	2369 ± 621	**0.007**
Carbohydrate (% of energy)	55.9 ± 6.5	56.0 ± 7.4	0.913
protein(% of energy)	13.2 ± 2.2	13.2 ± 2.5	0.924
fat(% of energy)	30.8 ± 6.5	30.8 ± 7.4	0.939
Fiber (g/1000 Kcal)	15.9 ± 6.5	16.8 ± 8.3	0.121
Healthy lifestyle score			< 0.001
Low (%)	10.4	50.2	
Middle (%)	39.6	42.2	
High (%)	50.0	7.6	
Alternate healthy eating index	55.1 ± 8.1	48.8 ± 7.7	**< 0.001**
***Alternate healthy eating index components***			
Vegetables (servings/d)	2.86 (1.95–3.97)	2.47 (1.63–3.40)	**0.001**
Fruit (servings/d)	2.60 (1.35–4.00)	2.57 (1.41–4.29)	0.877
Whole grains (servings/d)	1.75 (0.85–3.82)	2.13 (0.98–3.80)	0.860
Sugar-sweetened beverages and fruit juice (servings/d)	0.09 (0.04–0.15)	0.15 (0.06–0.32)	**< 0.001**
Nuts and legumes (servings/d)	0.27 (0.16–0.50)	0.28 (0.15–0.51)	0.537
Red/processed meat (servings/d)	0.60 (0.33–0.98)	0.87 (0.55–1.35)	**< 0.001**
Trans fatty acid (% of energy)	0.68 (0.51–0.96)	1.7 (0.88–2.36)	**< 0.001**
Long-chain (n-3) fats ( mg/d)	0.07 (0.03–0.13)	0.06 (0.03–0.12)	0.535
Polyunsaturated fatty acid (% of energy)	6.03 (4.80–7.55)	6.20 (4.84–7.63)	0.193
Sodium ( mg/d)	3425 (2450–5448)	3547 (2681–5213)	0.602

^*^ Data were represented as mean ± SD, or median (IQR 25–75) for continuous variables and percent for categorical variables

The characteristics of the study population according to tertiles of HLS have been reported in Table 2. The percentage of smoking, married individuals, and men in the highest tertile of HLS were lower than those in the lowest tertile of HLS (P < 0.05). Also, compared to participants in the lowest tertile of HLS, individuals in the highest tertile of HLS had lower levels of BMI and higher physical activity (P < 0.05). Findings on dietary intakes of participants according to tertiles of HLS showed that individuals in the highest tertile of HLS had higher consumption of vegetables, fruit, whole grains, nuts, and legumes, and a lower intake of sugar-sweetened beverages and fruit juice, red and processed meat, and TFA than those in the lowest tertile of HLS (P < 0.05). We did not observe a significant difference in other characteristics among participants according to tertiles of HLS.

**Table 2:** Study population characteristics according to tertiles of healthy lifestyle score ^*^

Variables	Tertiles of Healthy Lifestyle score			P-Value
	T1 (n = 160)	T1 (n = 273)	T3 (n = 242)	
**Demographic data**				
Age,(year)	38.6 ± 8.5	38.2 ± 8.6	37.6 ± 9.2	0.482
Men, (%)	55.0	48.4	40.1	**0.011**
Body mass index (Kg.m^2^)	30.5 ± 3.9	27.4 ± 3.7	23.7 ± 2.7	**< 0.001**
Physical activity ( MET/min/week)	802 ± 382	1423 ± 895	1861 ± 849	**< 0.001**
Smoking (yes, %)	11.9	3.3	0.0	**< 0.001**
Marital status (married, %)	88.1	87.9	76.3	**0.001**
Education level (Bachelor and higher, %)	46.2	47.6	46.3	0.942
Socio economic status (%)				0.118
Low (%)	25.0	28.6	29.8	
Middle (%)	33.1	38.5	41.3	
High (%)	41.9	33.0	28.9	
**Dietary intake**				
Energy intake(Kcal/d)	2250 ± 650	2273 ± 661	2292 ± 612	0.811
Carbohydrate (% of energy)	57.4 ± 7.1	57.0 ± 7.4	57.3 ± 7.2	0.702
protein(% of energy)	13.4 ± 2.7	13.7 ± 2.2	13.5 ± 2.2	0.401
fat(% of energy)	31.2 ± 6.9	31.7 ± 7.2	31.6 ± 6.6	0.772
Fiber (g/1000 Kcal)	16.9 ± 8.9	16.0 ± 6.9	15.7 ± 5.9	0.244
Healthy lifestyle score	0.95 ± 0.20	2.00 ± 0.00	3.27 ± 0.44	**< 0.001**
Alternate healthy eating index	46.4 ± 5.9	52.0 ± 8.1	56.8 ± 7.4	**< 0.001**
***Alternate healthy eating index components***				
Vegetables (servings/d)	2.34 (1.72–3.21)	2.80 (1.76–3.74)	2.97 (1.90–4.22)	**< 0.001**
Fruit (servings/d)	1.90 (1.25–3.22)	2.57 (1.18–4.26)	3.10 (1.78–4.42)	**0.001**
Whole grains (servings/d)	1.67 (0.72–3.28)	1.86 (0.85–3.96)	2.29 (1.01–3.96)	**< 0.001**
Sugar-sweetened beverages and fruit juice (servings/d)	0.03 (0.03–0.16)	0.03 (0.00–0.07)	0.02 (0.00–0.03)	**0.001**
Nuts and legumes (servings/d)	0.23 (0.12–0.38)	0.27 (0.16–0.50)	0.30 (0.17–0.54)	**< 0.001**
Red/processed meat (servings/d)	0.84 (0.53–1.34)	0.75 (0.43–1.08)	0.52 (0.29–0.91)	**0.016**
Trans fatty acid (% of energy)	1.25 (0.82–2.21)	0.87 (0.59–1.43)	0.65 (0.51–0.89)	**< 0.001**
Long-chain (n-3) fats ( mg/d)	0.06 (0.02–0.12)	0.07 (0.04–0.13)	0.06 (0.03–0.12)	0.861
Polyunsaturated fatty acid (% of energy)	6.02 (4.84–7.28)	5.91 (4.69–7.52)	6.38 (4.89–7.78)	0.781
Sodium ( mg/d)	3772 (2551–6457)	3419 (2575–5549)	3316 (2419–5099)	0.561

^*^ Data were represented as mean ± SD, or median (IQR 25–75) for continuous variables and percent for categorical variables

The OR of NAFLD across tertiles of AHEI and HLS is shown in Table 3. Based on the age and sex-adjusted model, the odds of NAFLD were decreased across tertiles of AHEI (OR: 0.18; 95%CI: 0.16–0.29, P_trend_<0.001) and HLS (OR: 0.03; 95%CI: 0.01–0.05, P_trend_<0.001). Also, in the multivariable model, after adjusting for age, sex, BMI (only for AHEI), smoking (only for AHEI), physical activity (only for AHEI), marital status, SES, and dietary intake of energy, the odds of NAFLD were decreased across tertiles AHEI (OR: 0.12; 95%CI: 0.06–0.24, P_trend_<0.001) and HLS (OR: 0.02; 95%CI: 0.01–0.04, P_trend_<0.001).

**Table 3:** The association between the alternate healthy eating index and healthy lifestyle index with odds of NAFLD

Indices	OR(95% CI) of NAFLD			P _trend_
	T1	T2	T3	
**Alternate healthy eating index**				
Median score	43.9	52.2	60.3	
Case/Total	111 / 225	77 / 225	37 / 225	
Crude model	1.00 (Ref)	0.52 (0.36–0.77)	0.20 (0.13–0.31)	< 0.001
Model 1*	1.00 (Ref)	0.50 (0.34–0.74)	0.18 (0.16–0.29)	< 0.001
Model 2^†^	1.00 (Ref)	0.35 (0.20–0.62)	0.12 (0.06–0.24)	< 0.001
**Healthy lifestyle score**				
Median score	1.00	2.00	3.00	
Case/Total	113 / 160	95 / 273	17 / 242	
Crude model	1.00 (Ref)	0.22 (0.14–0.34)	0.03 (0.02–0.05)	< 0.001
Model 1*	1.00 (Ref)	0.21 (0.13–0.32)	0.03 (0.01–0.05)	< 0.001
Model 2^‡^	1.00 (Ref)	0.20 (0.13–0.31)	0.02 (0.01–0.04)	< 0.001

^*^ Model 1: adjusted for age and sex

^†^ Model 2: adjusted for model 1 and body mass index, smoking, physical activity, marital status, socio-economic status, and dietary intake of energy

^‡^ Model 2: adjusted for model 1 and marital status, socio-economic status, and dietary intake of energy

## Discussion

The present study showed that a higher HLS score was may be associated with reduced odds of NAFLD, independent of potential confounding factors. Also, greater adherence to a diet with a high AHEI score is related to lower odds of NAFLD.

Recently, in various studies, the relationship between the HLS index, characterized by no smoking, no obesity, a healthy diet, and high physical activity, and the risk of cardiometabolic disorders has been investigated, the results of which have shown its strong predictive power in reducing these chronic diseases, including coronary artery disease [16], type 2 diabetes [33], metabolic syndrome [18], mortality [19]. Although our study is the first study in the MENA region, such as Iran to show the possible association of HLS with the reduction in risk of NAFLD, in Western societies as well as East Asian countries, the relationship of HLS with the risk of NAFLD has been investigated, which indicating interesting results. American Heart Association proposed a definition of cardiovascular health in 2010 (Life’s Simple 7, LS7) and updated it in 2022 (Life’s Essential 8, LE8) [34, 35]. This score, like the HLS score, uses AHEI as an indicator of nutritional status, which shows that AHEI is a suitable index for the study of cardiometabolic diseases. Consistent with our findings, they have demonstrated that a more favorable cardiovascular health score is associated with a lower risk of NAFLD [34, 35]. Also, a study recently conducted on the US population suggested that a higher HLS score was related to a lower risk of NAFLD and clinically significant fibrosis and can improve liver function [20], also, a cross-sectional study in early adulthood showed that a lifestyle with high HLS is inversely associated with hepatic steatosis index and fatty liver index [21]. Furthermore, two studies on the Chinese population [22, 23] reported that higher adherence to lifestyle with a high score of HLS may be associated with reduced risk of NAFLD. In line with the findings of previous studies, the results of our study showed that in the Iranian adult population, adherence to a healthy eating pattern, determined by the AHEI index, and having a healthy lifestyle based on a high HLS score can protect them against the occurrence of chronic diseases, such as NAFLD. The results of our study can also be interesting and important for researchers in another way because this study was conducted in the Iranian population as a country in the MENA region, which may have food habits and lifestyles different from Western societies. Therefore, our study confirms that the use of these dietary and lifestyle indicators in other populations, such as Asian societies, can appropriately predict the status of dietary patterns and lifestyles in their population.

Our findings on the association of AHEI and the risk of NAFLD are comparable with the results of some studies that have evaluated the role of AHEI as a major diet quality index in liver diseases such as NAFLD. In line with our study, Framingham Heart study reported that a higher score of AHEI was correlated with decreased fat accumulation in the liver and lower severity and risk of fatty liver in adult individuals, particularly in those with genetic risk factors for NAFLD [36]. In a multiethnic cohort study, following a high-quality diet, characterized by the higher score of HEI-2010 and AHEI-2010, reduced the risk of NAFLD [37]. Furthermore, Park et al. [38] revealed that a higher HEI-2015 score was negatively associated with NAFLD progression in ethnically different populations, but no association was found between the AHEI score with the risk of NAFLD. Notably, in NAFLD with cirrhosis, a significant inverse association was reported for AHEI in addition to DASH and HEI-2015, emphasizing that a high-quality diet was more beneficial to cirrhotic patients with NAFLD than for NAFLD patients without cirrhosis [38]. Therefore, the results of our study, as well as the previous evidence [10, 38], indicate that assessing the diet quality of individuals using the AHEI score as a healthy dietary index can help us to determine and identify the role of a healthy diet both alone or as important part of the HLS in predicting the risk of NAFLD.

The considerable ability of HLS in predicting the reduced odds of NAFLD can be explained based on the characteristics of its components. Higher adherence to a healthy diet is one of the most important components of HLS, which alone can play a very strong role in reducing the risk of this disease. We determined the quality of individuals’ diets based on the HEI index, which emphasizes a high intake of vegetables, fruit, whole grains, nuts and legumes, long-chain (n-3) fats, polyunsaturated fatty acid, and a low intake of sugar-sweetened beverages and fruit juice, red and processed meat, trans fatty acid, sodium. These above-mentioned food components can explain the role of HLS and AHEI in predicting the risk of NAFLD through possible mechanisms. Among AHEI components, nuts, vegetables, and dairy decrease the risk of NAFLD via high amounts of vitamins, minerals, and fibers [39]. Also, vegetables and fruits reduce the risk of NAFLD due to their high content of antioxidants, fiber, and phytochemicals [40]. Fibers control lipid levels and blood glucose, and phytochemicals and antioxidants are known as anti-inflammatory components that decrease hepatic steatosis [40]. NAFLD prevalence is associated with higher consumption of red meat and simple sugar [41]. High amounts of animal protein cause insulin resistance and glucose intolerance [42]. Also, red meats include saturated fatty acids that increase trans-10, cis-12 conjugated linoleic acid that promotes stress in the endoplasmic reticulum, and apoptosis which is connected to NAFLD progression [43]. Furthermore, heme-iron rich in meats induces oxidative stress that may lead to NAFLD [44]. High consumption of sweetened beverages and simple sugar increases the risk of NAFLD by increasing obesity, insulin resistance, lipid peroxidation, inflammatory reactions, and fat accumulation in the liver tissue [45]. Therefore, adherence to a diet with a high AHEI score in the form of a healthy lifestyle, one of the characteristics of which is the lower intake of red meat, simple sugar, and saturated fat, can play a notable role in reducing the risk of NAFLD.

Other lifestyle factor components, including smoking, physical activity, and obesity, may independently play a significant role in predicting liver disorders by interfering with different metabolic pathways. Smoking results in insufficient secretion of insulin and insulin resistance via ß-cell dysfunction, oxidative stress, inflammation, and increased level of C reactive protein (CRP) that may promote the risk of cardio-metabolic dysfunction [46, 47]. Physical activity improves insulin metabolism due to the up-regulation of insulin receptors in muscles leading to the beneficial delivery of glucose and insulin to muscles, reducing central obesity via negative energy balance, and increasing antioxidant capacity [48]. Obesity, a leading cause of NAFLD, is associated with hyperinsulinemia, impaired hepatic metabolism of glucose and free fatty acids, insulin resistance, and dyslipidemia, all of which are associated with an increased risk of NAFLD [40, 49]. Considering the individual impact of each lifestyle factor on NAFLD, determining a single index that shows the combined effect of important lifestyle components, including diet, physical activity, smoking, and obesity, could help us to accurately recognize the possible role of a healthy lifestyle in predicting the risk of chronic diseases, including NAFLD; Our results strongly indicated that a healthy lifestyle characterized by vigorous physical activity, no obesity, abstinence from smoking, and adhered to a healthy dietary pattern with high intake of fruits, legumes, vegetables, nuts and grains and low consumption of sweetened beverages and red and processed meats noticeably reduce the risk of NAFLD.

To the best of our knowledge, this is the first study that assessed the combined role of HLS in predicting NAFLD in the adult population. We used a reliable and valid physical activity and food frequency questionnaire to obtain the participants’ data. Furthermore, our analyses have adjusted for confounding factors to improve the reliability. Our study has some limitations. Due to our study design’s case-control nature, we could not discover a causal relationship between exposures and outcomes. Using FFQ to collect dietary intake data makes measurement errors inevitable; however, we tried to minimize this error using a validated questionnaire. For the detection of NAFLD in individuals, a biopsy of the liver and magnetic resonance imaging technique is the gold standard tests; however, in the current study, the ultrasonography test was used to diagnose NAFLD in participants; because using biopsy as an expensive diagnostic test has limitations and complications and MRI is also a high cost and less available test, but ultrasonography test is a noninvasive and easily accessible test that is applicable and reliable to detect NAFLD in individuals. Although our aim in this study was to investigate the cumulative effect of lifestyle factors in the form of HLS score, the issue that HLS does not provide any information on which lifestyle factor or the cluster of components has a greater predictive value can be considered as another limitation for our study. Finally, although the confounding effects of several confounders were controlled in analyses of data of the present study, our study design may not consider all potential confounders and the effects of some residual confounders may have occurred.

## Conclusions

In conclusion, our results showed that greater adherence to HLS, defined by normal BMI, healthy diet, high physical activity, and not smoking, are significantly associated with a reduced risk of NAFLD. Also, a diet with a high AHEI score independently is related to decreased odds of NAFLD. To confirm the evidence obtained from our study on the possible relationship between HLS and AHEI scores and the NAFLD risk, it is suggested to conduct more epidemiological studies, especially prospective cohort studies with a large sample size and a longer follow-up period.

## Data Availability

The datasets analyzed in the current study are available from the corresponding author on reasonable request.

## Abbreviations

AHEI: alternate healthy eating index
BMI: body mass index
CIs: confidence intervals
FCT: Food Composition Table
FFQ: food frequency questionnaire
IPAQ: International Physical Activity Questionnaire
METs: Metabolic Equivalents
MetS: metabolic syndrome
NAFLD: Nonalcoholic fatty liver disease
ORs: odds ratios
SD: Standard deviation
SES: socioeconomic status
SPSS: Statistical Package Software for Social Science
USDA: United States Department of Agriculture

## References

[1] Younossi ZM, Koenig AB, Abdelatif D, Fazel Y, Henry L, Wymer M. “Global epidemiology of nonalcoholic fatty liver disease-Meta-analytic assessment of prevalence, incidence, and outcomes,“ *Hepatology, vol* 64, no. 1, pp. 73–84. 10.1002/hep.28431 26707365

[2] Younossi Z, Anstee QM, Marietti M, Hardy T, Henry L, Eslam M et al. “Global burden of NAFLD and NASH: trends, predictions, risk factors and prevention,“ Nat Rev Gastroenterol Hepatol, *vol* 15, no. 1, pp. 11–20. 10.1038/nrgastro.2017.109 28930295

[3] Paik JM, Golabi P, Younossi Y, Mishra A, Younossi ZM. “Changes in the Global Burden of Chronic Liver Diseases From 2012 to 2017: The Growing Impact of NAFLD,“ *Hepatology, vol* 72, no. 5, pp. 1605-16. 10.1002/hep.31173 32043613

[4] Estes C, Razavi H, Loomba R, Younossi Z, Sanyal AJ. “Modeling the epidemic of nonalcoholic fatty liver disease demonstrates an exponential increase in burden of disease,“ *Hepatology, vol* 67, no. 1, pp. 123 – 33. 10.1002/hep.29466 PMC5767767 28802062

[5] Etminani R, Manaf Z, Shahar S, Azadbakht L, Adibi P. “Predictors of nonalcoholic fatty liver disease among middle-aged Iranians,“ Int J Prev Med, *vol* 11, no. 1, pp. 113-. 10.4103/ijpvm.IJPVM_274_19 PMC7554566 33088441

[6] Chaney A. “Obesity and Nonalcoholic Fatty Liver Disease,“ Nurs Clin North Am, *vol* 56, no. 4, pp. 543–52. 10.1016/j.cnur.2021.07.009 34749893

[7] Kim DV-ML, Li AA, Cholankeril G, Ahmed A. “Inadequate Physical Activity and Sedentary Behavior Are Independent Predictors of Nonalcoholic Fatty Liver Disease,“ *vol* 72, no. 5, pp. 1556–68. 10.1002/hep.31158 32012316

[8] Akhavan Rezayat A, Dadgar Moghadam M, Ghasemi Nour M, Shirazinia M, Ghodsi H, Rouhbakhsh Zahmatkesh MR et al. “Association between smoking and non-alcoholic fatty liver disease: A systematic review and meta-analysis,“ SAGE Open Med, *vol* 6, p. 2050312117745223. 10.1177/2050312117745223 PMC5788091 29399359

[9] Hassani Zadeh SMA, Hosseinzadeh M. “Relationship between dietary patterns and non-alcoholic fatty liver disease: A systematic review and meta-analysis,“ *vol* 36, no. 6, pp. 1470–8. 10.1111/jgh.15363 33269500

[10] Mirashrafi S, Kafeshani M, Hassanzadeh A, Entezari MH. “Is any association between alternate healthy eating index (AHEI) with lipid profile and liver enzymes? A cross-sectional Study,“ J Diabetes Metab Disord, *vol* 20, no. 2, pp. 1537–44. 10.1007/s40200-021-00898-w PMC8630323 34900806

[11] McCullough ML, Willett WC. “Evaluating adherence to recommended diets in adults: the Alternate Healthy Eating Index,“ Public Health Nutr, *vol* 9, no. 1a, pp. 152–7. 10.1079/phn2005938 16512963

[12] Vilar-Gomez E, Nephew LD, Vuppalanchi R, Gawrieh S, Mladenovic A, Pike F et al. “High‐quality diet, physical activity, and college education are associated with low risk of NAFLD among the US population,“ Hepatology, *vol* 75, no. 6, pp. 1491–506. 10.1002/hep.32207 34668597

[13] Nechuta SJ, Shu X-O, Li H-L, Yang G, Xiang Y-B, Cai H et al. “Combined Impact of Lifestyle-Related Factors on Total and Cause-Specific Mortality among Chinese Women: Prospective Cohort Study,“ PLoS Med, *vol* 7, no. 9, p. e1000339. 10.1371/journal.pmed.1000339 PMC2939020 20856900

[14] Prochaska JJ, Velicer WF, Nigg CR, Prochaska JO. “Methods of quantifying change in multiple risk factor interventions,“ Prev Med, *vol* 46, no. 3, pp. 260–5. 10.1016/j.ypmed.2007.07.035 PMC2288581 18319099

[15] Chiuve SE, McCullough ML, Sacks FM, Rimm EB. “Healthy lifestyle factors in the primary prevention of coronary heart disease among men: benefits among users and nonusers of lipid-lowering and antihypertensive medications,“ *Circulation, vol* 114, no. 2, pp. 160-7. 10.1161/CIRCULATIONAHA.106.621417 16818808

[16] Khera AV, Emdin CA, Drake I, Natarajan P, Bick AG, Cook NR et al. “Genetic Risk, Adherence to a Healthy Lifestyle, and Coronary Disease,“ N Engl J Med, *vol* 375, no. 24, pp. 2349–58. 10.1056/NEJMoa1605086 PMC5338864 27959714

[17] Farhadnejad H, Teymoori F, Asghari G, Mokhtari E, Mirmiran P, Azizi F. “The higher adherence to a healthy lifestyle score is associated with a decreased risk of type 2 diabetes in Iranian adults,“ BMC Endocr Disord, *vol* 22, no. 1, p. 42. 10.1186/s12902-022-00961-4 PMC8855558 35177061

[18] Mirmiran PFH, Teymoori F, Parastouei K, Azizi F. “The higher adherence to healthy lifestyle factors is associated with a decreased risk of metabolic syndrome in Iranian adults,“ *vol* 47, no. 1, pp. 57–67. 10.1111/nbu.12537 36045083

[19] Patel YR, Gadiraju TV, Gaziano JM, Djoussé L. “Adherence to healthy lifestyle factors and risk of death in men with diabetes mellitus: The Physicians’ Health Study,“ Clin Nutr, *vol* 37, no. 1, pp. 139–43. 10.1016/j.clnu.2016.11.003 PMC5513764 27866759

[20] Zhu Y, Yang H, Liang S, Zhang H, Mo Y, Rao S et al. “Higher Adherence to Healthy Lifestyle Score Is Associated with Lower Odds of Non-Alcoholic Fatty Liver Disease,“ *Nutrients, vol* 14, no. 21, p. 4462. 10.3390/nu14214462 PMC9657000 36364725

[21] Schnermann ME, Schulz C-A, Perrar I, Herder C, Roden M, Alexy U et al. “A healthy lifestyle during adolescence was inversely associated with fatty liver indices in early adulthood: findings from the DONALD cohort study,“ Br J Nutr, *vol* 129, no. 3, pp. 513–22. 10.1017/S0007114522001313 35492013

[22] Deng Y-y, Zhong Q-w, Zhong H-l, Xiong F, Ke Y-b, Chen Y-m. “Higher Healthy Lifestyle Score is associated with lower presence of non-alcoholic fatty liver disease in middle-aged and older Chinese adults: a community-based cross-sectional study,“ Public Health Nutr, *vol* 24, no. 15, pp. 5081–9. 10.1017/S1368980021000902 PMC11082827 33634772

[23] Ling Z, Zhang C, He J, Ouyang F, Qiu D, Li L et al. “Association of Healthy Lifestyles with Non-Alcoholic Fatty Liver Disease: A Prospective Cohort Study in Chinese Government Employees,“ *Nutrients, vol* 15, no. 3, p. 604. 10.3390/nu15030604 PMC9921275 36771311

[24] Mokhtari E, Farhadnejad H, Salehi-Sahlabadi A, Najibi N, Azadi M, Teymoori F et al. “Spinach consumption and nonalcoholic fatty liver disease among adults: a case–control study,“ BMC Gastroenterol, *vol* 21, no. 1, p. 196. 10.1186/s12876-021-01784-8 PMC8088717 33933019

[25] Esmaillzadeh A, Kimiagar M, Mehrabi Y, Azadbakht L, Hu FB. Willett WCJTAjocn. “Dietary patterns, insulin resistance, and prevalence of the metabolic syndrome in women,“ *vol* 85, no. 3, pp. 910-8. 10.1093/ajcn/85.3.910 17344515

[26] Ghaffarpour M, Houshiar-Rad A, Kianfar HJTNOK. “The manual for household measures, cooking yields factors and edible portion of foods,“ *vol* 7, p. 213.

[27] Bowman SA, Friday JE, Moshfegh AJJUDoA. “MyPyramid Equivalents Database, 2.0 for USDA survey foods, 2003–2004: documentation and user guide.“.

[28] Moghaddam MB, Aghdam FB, Jafarabadi MA, Allahverdipour H, Nikookheslat SD, Safarpour S. “The Iranian Version of International Physical Activity Questionnaire (IPAQ) in Iran: content and construct validity, factor structure, internal consistency and stability,“ World Appl Sci J, *vol* 18, no. 8, pp. 1073–80.

[29] Ainsworth BE, Haskell WL, Whitt MC, Irwin ML, Swartz AM, Strath SJ et al. “Compendium of physical activities: an update of activity codes and MET intensities,“ Med Sci Sports Exerc, *vol* 32, no. 9; SUPP/1, pp. S498–S504. 10.1097/00005768-200009001-00009 10993420

[30] Lee PH, Macfarlane DJ, Lam TH, Stewart SM. “Validity of the international physical activity questionnaire short form (IPAQ-SF): A systematic review,“ Int J Behav Nutr Phys activity, *vol* 8, no. 1, pp. 1–11. 10.1186/1479-5868-8-115 PMC3214824 22018588

[31] Garmaroudi GR, Moradi A. “Socio-economic status in Iran: a study of measurement index,“ Payesh (Health Monitor), *vol* 9, no. 2, pp. 137–44.

[32] Chiuve SE, Fung TT, Rimm EB, Hu FB, McCullough ML, Wang M et al. “Alternative dietary indices both strongly predict risk of chronic disease,“ J Nutr, *vol* 142, no. 6, pp. 1009–18. 10.3945/jn.111.157222 PMC3738221 22513989

[33] Farhadnejad H, Teymoori F, Asghari G, Mokhtari E, Mirmiran P, Azizi F. “The higher adherence to a healthy lifestyle score is associated with a decreased risk of type 2 diabetes in Iranian adults,“ BMC Endocr Disorders, *vol* 22, no. 1, pp. 1–12. 10.1186/s12902-022-00961-4 PMC8855558 35177061

[34] Wang L, Yi J, Guo X, Ren X. “Associations between life’s essential 8 and non-alcoholic fatty liver disease among US adults,“ J Translational Med, *vol* 20, no. 1, pp. 1–10. 10.1186/s12967-022-03839-0 PMC9789599 36564799

[35] Oni E, Ogunmoroti O, Allen N, A-Mallah MH, Blankstein R, Martin SS et al. “Life’s simple 7 and nonalcoholic fatty liver disease: the multiethnic study of atherosclerosis,“ Am J Med, *vol* 134, no. 4, pp. 519–25. 10.1016/j.amjmed.2020.09.023 33285128

[36] Ma J, Hennein R, Liu C, Long MT, Hoffmann U, Jacques PF et al. “Improved diet quality associates with reduction in liver fat, particularly in individuals with high genetic risk scores for nonalcoholic fatty liver disease,“ *Gastroenterology, vol* 155, no. 1, pp. 107 – 17. 10.1053/j.gastro.2018.03.038 PMC6035111 29604292

[37] Maskarinec G, Lim U, Jacobs S, Monroe KR, Ernst T, Buchthal SD et al. “Diet quality in midadulthood predicts visceral adiposity and liver fatness in older ages: the Multiethnic Cohort Study,“ *Obesity, vol* 25, no. 8, pp. 1442-50. 10.1002/oby.21868 PMC5604249 28745024

[38] Park SY, Noureddin M, Boushey C, Wilkens LR, Setiawan VW. “Diet Quality Association with Nonalcoholic Fatty Liver Disease by Cirrhosis Status: The Multiethnic Cohort,“ Curr Dev Nutr, *vol* 4, no. 3, p. nzaa024. 10.1093/cdn/nzaa024 PMC7066377 32190810

[39] Dehghanseresht N, Jafarirad S, Alavinejad SP, Mansoori A. “Association of the dietary patterns with the risk of non-alcoholic fatty liver disease among Iranian population: a case-control study,“ Nutr J, *vol* 19, no. 1, p. 63. 10.1186/s12937-020-00580-6 PMC7329390 32605646

[40] Mirmiran P, Amirhamidi Z, Ejtahed H-S, Bahadoran Z, Azizi F. “Relationship between diet and non-alcoholic fatty liver disease: a review article,“ Iran J public health, *vol* 46, no. 8, p. 1007. PMC5575379 28894701

[41] Zelber-Sagi S, Nitzan-Kaluski D, Goldsmith R, Webb M, Blendis L, Halpern Z et al. “Long term nutritional intake and the risk for non-alcoholic fatty liver disease (NAFLD): a population based study,“ J Hepatol, *vol* 47, no. 5, pp. 711–7. 10.1016/j.jhep.2007.06.020 17850914

[42] Linn T, Geyer R, Prassek S, Laube H. “Effect of dietary protein intake on insulin secretion and glucose metabolism in insulin-dependent diabetes mellitus,“ J Clin Endocrinol Metabolism, *vol* 81, no. 11, pp. 3938–43. 10.1210/jcem.81.11.8923841 8923841

[43] Wei Y, Wang D, Pagliassotti MJ. “Saturated fatty acid-mediated endoplasmic reticulum stress and apoptosis are augmented by trans-10, cis-12-conjugated linoleic acid in liver cells,“ Mol Cell Biochem, *vol* 303, no. 1, pp. 105–13. 10.1007/s11010-007-9461-2 17426927

[44] Alla V, Bonkovsky HL, editors. Iron in nonhemochromatotic liver disorders. Seminars in liver disease; 2005: Copyright© 2005 by Thieme Medical Publishers, Inc., 333 Seventh Avenue, New …. 10.1055/s-2005-923317 16315139

[45] Asgari-Taee F, Zerafati-Shoae N, Dehghani M, Sadeghi M, Baradaran HR, Jazayeri S. “Association of sugar sweetened beverages consumption with non-alcoholic fatty liver disease: a systematic review and meta-analysis,“ Eur J Nutr, *vol* 58, no. 5, pp. 1759–69. 10.1007/s00394-018-1711-4 29761318

[46] Stadler M, Tomann L, Storka A, Wolzt M, Peric S, Bieglmayer C et al. “Effects of smoking cessation on b-cell function, insulin sensitivity, body weight, and appetite,“ Eur J Endocrinol, *vol* 170, no. 2, pp. 219–27. 10.1530/EJE-13-0590 24179100

[47] van der Vaart H, Postma DS, Timens W, Ten Hacken NH. “Acute effects of cigarette smoke on inflammation and oxidative stress: a review,“ *Thorax, vol* 59, no. 8, pp. 713 – 21. 10.1136/thx.2003.012468 PMC1747102 15282395

[48] Weinstein AR, Sesso HD, Lee IM, Cook NR, Manson JE, Buring JE et al. “Relationship of physical activity vs body mass index with type 2 diabetes in women,“ JAMA, *vol* 292, no. 10, pp. 1188–94. 10.1001/jama.292.10.1188 15353531

[49] Al Rifai M, Silverman MG, Nasir K, Budoff MJ, Blankstein R, Szklo M et al. “The association of nonalcoholic fatty liver disease, obesity, and metabolic syndrome, with systemic inflammation and subclinical atherosclerosis: the Multi-Ethnic Study of Atherosclerosis (MESA),“ *Atherosclerosis, vol* 239, no. 2, pp. 629 – 33. 10.1016/j.atherosclerosis.2015.02.011 PMC4406399 25683387

